# Performance Assessment of Thermoelectric Generators with Application on Aerodynamic Heat Recovery

**DOI:** 10.3390/mi12111399

**Published:** 2021-11-14

**Authors:** Xiaodong Jia, Shifa Fan, Zhao Zhang, Hongbiao Wang

**Affiliations:** 1 High Speed Aerodynamics Institute, China Aerodynamics Research and Development Center, Mianyang 621000, China; zhangzhao04@tsinghua.org.cn; 2 Key Laboratory of Mechanics on Western Disaster and Environment with the Ministry of Education, College of Civil Engineering and Mechanics, Lanzhou University, Lanzhou 730000, China; fanshf17@lzu.edu.cn; 3 Department of Energy and Power Engineering, Tsinghua University, Beijing 100084, China; hb-wang15@mails.tsinghua.edu.cn

**Keywords:** vehicle, aerodynamic heat, thermoelectric generators, energy conversion

## Abstract

Based on thermoelectric generators (TEGs), an aerodynamic heat energy recovery system for vehicle is proposed. A mathematical model describing the energy conversion law of the system is established, and the integrated calculation method which combined aerodynamic heating and thermoelectric (TE) conversion is given. Furthermore, the influences of the typical flight Mach number, flight altitudes and the length of TE legs on the energy conversion behavior of energy recovery systems are investigated. The performance of the energy recovery system is analyzed and evaluated. The results show that, the decrease of flight altitude and the increase of Mach number will obviously improve the performance of the heat energy recovery system with TEGs. The increase of leg length will increase the temperature of the hot end of TEGs and reduce the heat absorbed at the hot end. When the external load, Mach number and flight altitude is fixed, there exists an optimal length of legs corresponding to the maximum output power and maximum conversion efficiency of the system. The results will have significant positive impact on thermal protection and management of supersonic/hypersonic vehicles.

## 1. Introduction

When the vehicle is flying at supersonic speed or hypersonic speed, the surrounding air is strongly compressed and frictioned on the surface of the body, which partially converts the kinetic energy of the vehicle into thermal energy, causing the surface temperature of the vehicle to rise rapidly. The resulting thermal protection problem has always been one of the key technical issues for the supersonic/hypersonic vehicles. Heat is a kind of energy. Recycling and utilizing heat, such as establishing a stable conversion of heat energy into electrical energy, can improve the performance of the vehicle to a certain extent and alleviate the pressure of thermal protection. Traditional energy conversion devices mostly adopt dynamic conversion. Although the conversion efficiency is high, it has a certain impact on its structural reliability due to its moving parts. Thermoelectric (TE) generator is a new type of energy conversion device. Based on the electronic transport of TE functional materials [1], it can directly realize the mutual conversion between thermal energy and electrical energy, with the advantages of small size, light weight, no moving parts, no noise, no pollution, long service life, high reliability [2,3,4,5]. It has broad application prospects in space exploration, solar energy, automobile industry, medical treatment and many other fields [6,7,8,9,10,11].

One difference between thermoelectric generators (TEGs) and conventional energy conversion devices is the variety of available heat sources. For example, Liu et al. [12] designed a radioisotope TE generator that meets the needs of low-power power supplies for deep space explorer and introduced the relationship between the output performance and the structure size, the heat source power of the TEGs. Ostrufk et al. [13] introduced a solar TE generator applied to CubeSat and the generation capacity is analyzed for different positioning configurations of the TE generator relative to each CubeSat surface. Lamba et al. [14] established the theoretical model of centralized solar TEGs, and analyzed the influence of solar concentration, input current, thermocouple number and load resistivity on the output power, energy and exergy efficiency of the power generation system. Meng et al. [15] proposed a waste heat recovery system for automobile exhaust based on TEGs, focusing on the impact of the temperature difference environment along the airflow direction of the exhaust pipe and the number of TE units on the performance of the TE system. Kim et al. [16] designed a wearable electrocardiography system based on self-powered body temperature, which uses wearable TEGs as energy source. Zhang et al. [17] have proposed a new hybrid system based on thermoelectric generators, thermoelectric coolers and solid oxide fuel cells, they found that the power density and efficiency of the proposed system allow 2.3% and 4.6% larger than that of the stand-alone solid oxide fuel cells, respectively. A novel compact heat sink module is proposed by Wiriyasart et al. [18], the module uses water as the loop fluid, and thermoelectric devices are added to it, thereby improving the temperature difference between the cold and hot sides and the output power. In addition, they also did some research on thermoelectric coolers [19,20,21]. The one-dimensional energy equilibrium approach is used by Liu et al. [22] to study the performance of variable cross-section TEGs, their results showed that compared with the traditional thermoelectric leg, the variable cross section thermoelectric leg can always improve the maximum conversion efficiency of TEGs. It can also be seen from the above literature that the structure size of TEGs has a great influence on their thermoelectric performance.

Meanwhile, for supersonic/hypersonic vehicles, the aerodynamic thermal environment creates the basic conditions for the application of TE functional materials and TEGs. Cheng et al. [23] presented a power generation scheme based on multistage thermoelectric generator for hypersonic vehicle, they optimized the geometry and operating parameters of the TEGs for the higher output power and conversion efficiency. In addition, Single-stage and two-stage TEG models with combustion heat dissipation as the heat source are established by Cheng et al. [24] to predict and compare the power generation performance of hypersonic vehicles at different inlet temperatures of heating channels. In the above research, the combustion heat in the aircraft engine is directly used as the heat source, and the coolant is used as the cold source [24]. Which increase the cost and weight of the entire generator system. However, if the natural environmental temperature difference between the cabin and the fuselage during flight is used to provide a working environment for TEGs. It would reduce the weight and complexity of the entire generator system and provides a new idea for TEGs to be used as emergency power for supersonic aircraft. Therefore, it is very necessary to study the performance of TEGs in this environment.

Based on the temperature difference environment between the fuselage of the vehicle and the internal equipment cabin, a novel system for recovering aerodynamic heat energy of the vehicle by means of a system-based TEG is proposed. A mathematical model to describe the aero-thermal and thermo-electric coupling effects of the energy recovery system is established. The aero-heating and TE conversion behaviors of the energy recovery system are studied, and the influences of typical flight conditions, geometric parameters of TE structure on the TE conversion performance of the energy recovery system are discussed, the effectiveness of the energy recovery system is also evaluated. Finally, some conclusions which may help to recover aerodynamic waste heat of the vehicle are given.

## 2. Mathematical Model of Energy Recovery System

### 2.1. Scheme of Energy Recovery System

**Figure 1:**
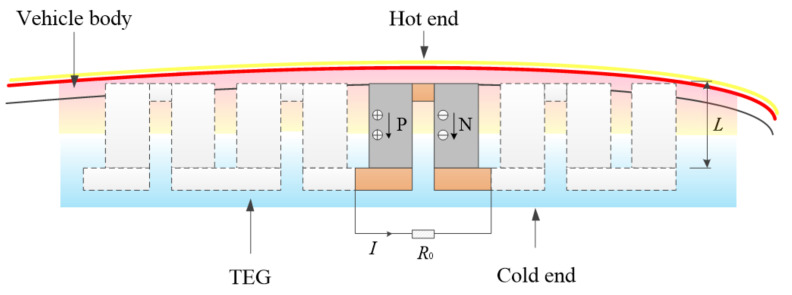
Schematic diagram of aerodynamic heat energy recovery system for vehicle.

Figure 1 presents the scheme of aerodynamic heat energy recovery system for vehicle. The P-type and N-type TE materials are arranged vertically and connected through the conductive layer material to form a single P-N thermocouple. The hot end of the thermocouple is arranged on the inner surface of thermal protection material layer of the vehicle, or embedded in the opening of the protective material, and the cold end is placed in the equipment cabin. The vehicle flies at high speed in the atmosphere for a long time, and continuously forms a high-speed airflow on the skin surface of the vehicle body. The high-speed airflow molecules collide with the body to generate a large amount of continuous and stable heat, which provides a heat source condition for the TE conversion of the energy recovery system. The electronic equipment compartment inside the vehicle has a certain storage space, which can solve the arrangement of thermoelectric devices. Due to the severe temperature requirements in the cabin, the corresponding refrigeration environment provides a cold source for the energy recovery system. 

The system works as follows: when the TEG obtains aerodynamic heat from the skin surface and forms a temperature difference at both ends of the TE legs, the carriers (electrons and holes) distributed evenly in TE functional materials begin to drift. The holes in p-leg and the electrons in n-leg are transported from the hot end to the cold end, resulting in a Seebeck potential difference at the cold ends of the TE legs, which forms a loop after being closed by an external load resistor (such as an electronic device), thus the energy recovery system collects thermal energy and directly converts it to electrical energy. In order to obtain higher output electric power, multiple groups of P-N thermocouple structures can be designed in electrical series and thermal parallel.

In order to simplify the problem studied in this paper, the following assumptions were adopted:

(1) Since the surface area of the vehicle skin structure is large, and the size of the TEG is small, the place where the TEG is placed on the fuselage can be regarded as a flat structure.
(2) Compressible laminar boundary layer flow with approximately zero pressure gradient on the surface of the vehicle
(3) The TEG directly obtains heat through the skin of the body, that is, the temperature of the hot end of the TEG is considered to be equal to the wall temperature of the equivalent flat structure of the body.
(4) The side wall of the TEG is wrapped with an adiabatic material to reduce heat loss. The heat exchange between the TE legs and the surrounding structure is not considered in the calculation.
(5) In order to reduce the factors that need to be considered simultaneously in the study, the temperature at the cold end of the TEG is the ambient temperature in the electronic compartment, assumed to be a fixed temperature value.
(6) The energy recovery system is in a stable operating environment.

### 2.2. Aero-Thermal and Thermo-Electric Coupling Governing Equations

The flow in the boundary layer of the vehicle equivalent flat structure can be obtained by solving the compressible laminar boundary layer equation, so as to determine the heat flow and wall temperature on the vehicle surface due to aerodynamic thermal effect. The flow governing equations are:

(1) $$\left\{\begin{array}{l} \frac{\partial }{\partial x}\left(\rho_{a}^{}u\right)+\frac{\partial }{\partial y}\left(\rho_{a}^{}v\right)=0\\ \rho_{a}^{}u\frac{\partial u}{\partial x}+\rho_{a}^{}v\frac{\partial u}{\partial y}=\frac{\partial }{\partial y}\left(\mu \frac{\partial u}{\partial y}\right)\\ \rho_{a}^{}u\cdot C_{p}^{}\frac{\partial T}{\partial x}+\rho_{a}^{}v\cdot C_{p}^{}\frac{\partial T}{\partial y}=\frac{\partial }{\partial y}\left(\lambda_{a}^{}\frac{\partial T}{\partial y}\right)+\mu {\left(\frac{\partial u}{\partial y}\right)}_{}^{2}\\ p_{a}=\rho_{a}^{}R_{a}T=\mathrm{const} \end{array}\right.$$

In which, *u* and *v* represent the flow velocity; *ρ*_a_, *p*_a_, *C*_p_, *μ*, R_a_ and *λ*_a_, respectively, represent airflow density, airflow pressure, gas isobaric specific heat capacity, air viscosity coefficient, gas constant and air thermal conductivity.

The heat flux density obtained by the TEG through the skin of the vehicle body is:

(2) $$q_{W}^{}=-{\left.\lambda_{a}\frac{\partial T}{\partial y}\right|}_{W}^{}$$

In TE analysis, the energy conservation equation and the charge continuity equation are:

(3) $$\left\{\begin{array}{l} \nabla \cdot q=\dot{q}\\ \nabla \cdot J=0 \end{array}\right.$$

Here, $\dot{q}=J\cdot E$ represents the internal heat source, including the electric power spent on Joule heating and on work against the Seebeck field. 

The constitutive equations describing the TE coupling behavior of TE functional materials are as follows:

(4) $$\left\{\begin{array}{l} q=T[\alpha ]J-[\lambda ]\nabla T\\ J=[\sigma ](E-[\alpha ]\nabla T) \end{array}\right.$$

In the equations, $\left[\alpha \right]$ is the Seebeck coefficient matrix, T is absolute temperature, $\left[\lambda \right]$ and $\left[\sigma \right]$ are thermal conductivity and the electric conductivity matrices, respectively. Moreover, J and E are the current density and electric potential vector, respectively. The electric field ***E*** can be obtained E=−∇φ by scalar potential. The first term on the right side of the heat flux equation of the Equation (4) represents the Peltier effect, and the second term represents the Fourier heat conduction effect. The first term on the right side of the current density equation represents ohm’s law, and the second term represents the Seebeck effect. If [*α*] = 0, there is no TE coupling effect, and the above formula can be reduced to the general form of the heat and electricity transport equations.

According to Equations (1)–(4), the governing equations of aero-thermal and thermo-electric coupling effects representing energy recovery system can be obtained.

### 2.3. Boundary Conditions

In aerodynamic heating analysis, flight altitude *h* should be given to calculate the gas flow temperature, pressure and thermophysical parameters of gas through the standard atmosphere model $f_{atm}^{}\left(h\right)$. The flight *Ma* number is given to calculate the incoming flow speed and Reynolds number Re. The boundary conditions of incoming flow are:

(5) (T∞,p∞,μ∞,λ∞)=fatm(h)u∞=u(Ma,T∞)Re=ρ∞u∞lμ∞

In the equations, *T*_∞_, *p*_∞_, *μ*_∞_, *λ*_∞_, *u*_∞_, *ρ*_∞_ and *l* are, respectively, the temperature, pressure, viscosity coefficient, thermal conductivity, velocity, density and reference length of the incoming flow under given flight conditions.

In TE analysis, the temperature of the cold end of the TEG is the ambient temperature in the cabin. The temperature of the hot end of the TEG is equal to the wall temperature of the boundary layer of the equivalent flat structure of vehicle. The heat absorbed from the flight environment is equal to the aerodynamic heat generated at the installation site of the energy recovery system of the vehicle. The thermal boundary conditions of the TEG are:

(6) $$\left\{\begin{array}{l} T_{C}=T_{0}\\ Q_{H}=Q_{W} \end{array}\right.$$

The electric potential of the cold end of p-leg is set as the reference electric potential, and the electric boundary conditions of the TEG can be expressed as:

(7) $$\left\{\begin{array}{l} \varphi_{p,C}=0\\ J_{n,C}=I/A_{n,C} \end{array}\right.$$

**Figure 2:**
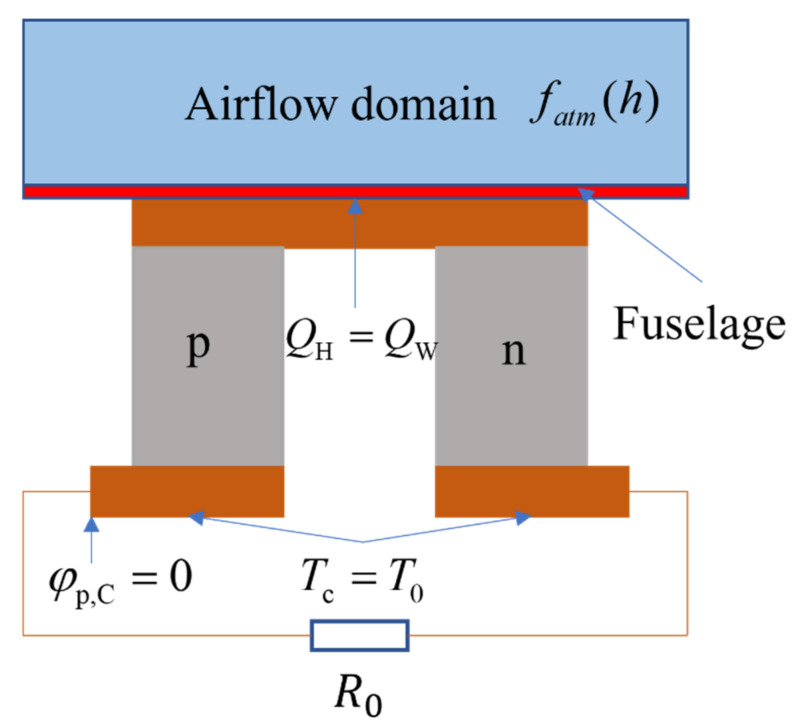
Schematic diagram of the boundary conditions.

In the equations, ***J****_n,C_* represents the current density of the cold end of n-leg, and *A_n,C_* represents the cross-sectional area of n-leg. The relevant boundary conditions are shown in Figure 2.

### 2.4. Solution Method

Under the assumption of laminar flow, the aerodynamic heating governing equation can be transformed into a system of ordinary differential equations by introducing the Illingworth transform and a flow function.

In the TE coupling governing equations, the physical parameters of TE materials have strong temperature dependence, and the governing equations are nonlinear, so it is difficult to obtain accurate solutions. The finite element method can be used to solve the governing equations numerically.

Given a current value *I* and temperature value *T*_C_ at the cold end of the TEG, the potential distribution, temperature distribution, temperature *T*_H_ at the hot end and absorbed heat flow *Q*_H_ of the TEG can be obtained by solving the governing equations of aero-thermal and thermo-electric coupling effects jointly. Then, the output power and TE conversion efficiency can be obtained through analytical calculation, namely:

(8) $$\left\{\begin{array}{l} P=I^{2}R_{0}\\ \eta =\frac{P}{Q_{H}} \end{array}\right.$$

The aero-thermal and thermo-electric coupling behaviors of the energy recovery system are numerically solved and analyzed with Matlab (MathWorks, Inc., Natick, MA, USA) and ANSYS (ANSYS Inc., Canonsburg, PA, USA) simulation tools.

## 3. Results and Discussion

**Figure 3:**
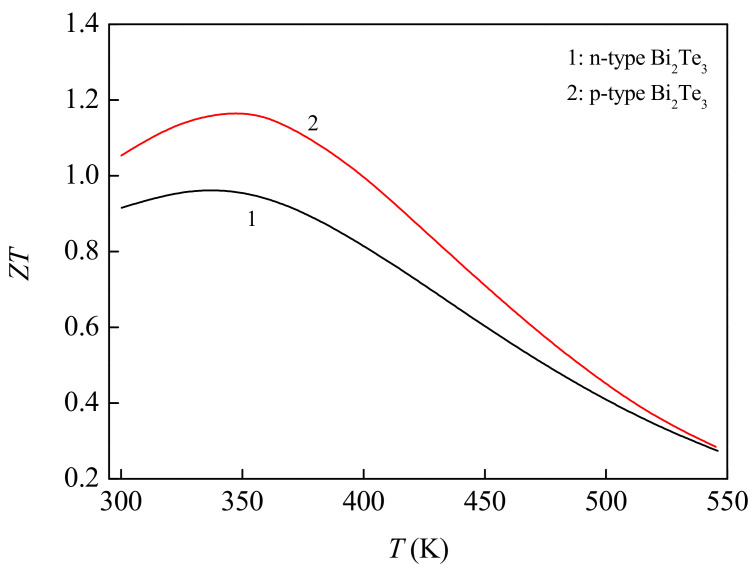
Figures of merit of Bi_2_Te_3_ materials [25].

This paper studies the effects of typical flight Mach number, flight altitude and length of TE legs on the performance of aerodynamic heat energy recovery system for vehicle. In the calculations, the selection of TE functional materials directly determines the performance of the system. The TE functional materials selected are Bi_2_Te_3_ alloys [25], whose dimensionless figures of merit *ZT* are shown in Figure 3. The applicable temperature range of this kind of material is 300~550 K, which is recognized as a TE functional material with excellent performance at low temperature. In the design of the vehicle, the reference length of the vehicle is set at *l* = 0.1 m, the flight Mach number is set at *Ma* = 3, 4, 5 and 6, and the flight altitude is set at *h* = 25, 30 and 35 km. The internal electronic equipment compartment of the vehicle is assumed to be at room temperature, *T*_0_ = 300 K. During the numerical simulation, the temperature at the cold end of the energy recovery system is set as *T*_C_ = 300 K, and the external load resistance of the energy recovery system is set as *R*_0_ = 0.01 Ω. Based on the preliminary exploration of the change of output performance with the geometric parameters of TEG in the early stage and taking into account the allowable space of the fuselage, the design reference range of the length of TE legs is to be set as 1~90 mm, and the cross-sectional area of TE legs is set as 10 × 10 mm^2^.

In order to verify the grid independence of the numerical model, the output power of thermoelectric system under three grid systems is calculated under the same boundary conditions ($T_{h}=400\;K,\;T_{c}=300\;K,{\;R}_{0}=0.01\;\Omega$). The sizes of the three grids are 1 mm, 0.5 mm and 0.4 mm, respectively, the corresponding output power of the device are 0.08718 W, 0.08716 W and 0.08716 W, respectively. The output power under the latter two grid systems is nearly equal. Finally, to reduce the cost of calculation and ensure the accuracy of the numerical model, the grid system with a 0.5 mm grid size was selected as the grid system for subsequent calculation.

**Figure 4:**
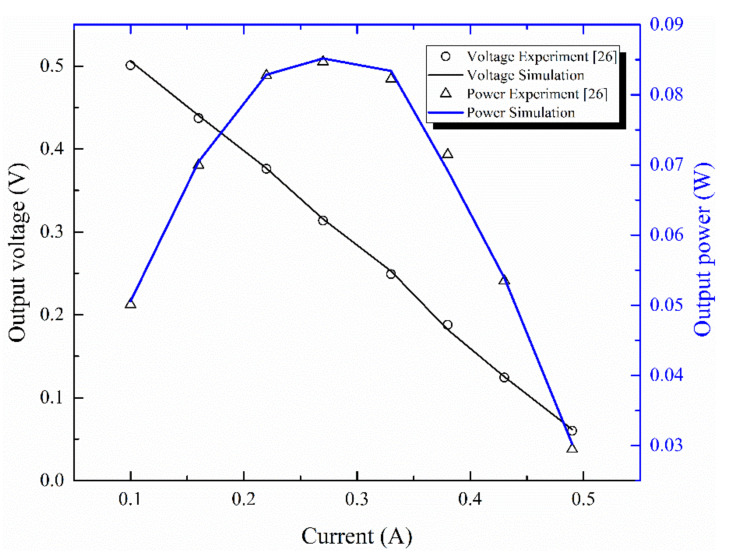
Comparison of the simulation results in this paper with the experimental data of Hsu et al. [26] ($T_{h}=310\;K$, $T_{C}=300\;K$).

To further verify the correctness of the model, the simulation results in this paper are first compared with the experimental data [26], as shown in Figure 4, where the material parameters and operating conditions involved are the same as the reference [26], and the influence of electrical contact resistance and contact thermal resistance [27] are taken into account. As can be seen from the figure, the simulation results are in good agreement with the experimental data [26], and the error is not more than 5%. Which proves the correctness and rationality of our numerical results.

**Figure 5:**
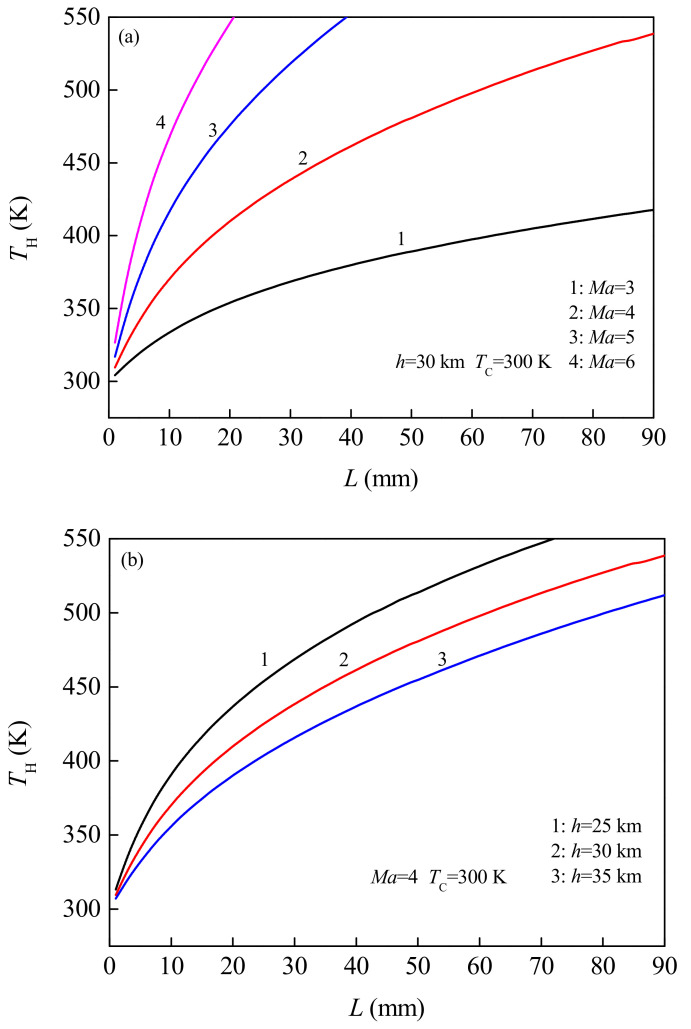
Variation of hot end temperature of TEG changing with the length of the TE leg under different flight Mach numbers (**a**) and flight altitudes (**b**).

In order to determine the effective design range of the TE legs in the energy recovery system, Figure 5 shows the variation rule of *T*_H_ (i.e., wall temperature of vehicle) of the hot end of the TEG changing with the length *L* of the TE legs under the conditions of typical flight Mach number *Ma* and flight altitude *h*. As can be seen from Figure 5a, for flight conditions of *Ma* = 6, *h* = 30 km, when the length of the TE leg is greater than 21 mm, the temperature of the hot end is more than 550 K, which is beyond the applicable temperature range of TE materials. Therefore, the effective design range of TE leg length here is 1~21 mm. For flight conditions of *Ma* = 5, *h* = 30 km, the effective design range of TE leg length is 1~40 mm. According to Figure 5b, for flight conditions *Ma* = 4 and *h* = 25 km, the effective design range is 1~74 mm. It can also be seen from the Figure that with the increase of the length of the TE legs, the temperature of the hot end increases continuously. In the given reference value of flight Mach number, the temperature value of the hot end corresponding to the high Mach number is significantly higher than that corresponding to the low Mach number (Figure 5a). Since the higher the Mach number, the more intense the friction between the vehicle fuselage and the airflow, the more significant the aerodynamic heating effect. In the given reference value of flight altitude, the lower the flight altitude is, the higher the temperature value of the hot end surface is (Figure 5b). 

**Figure 6:**
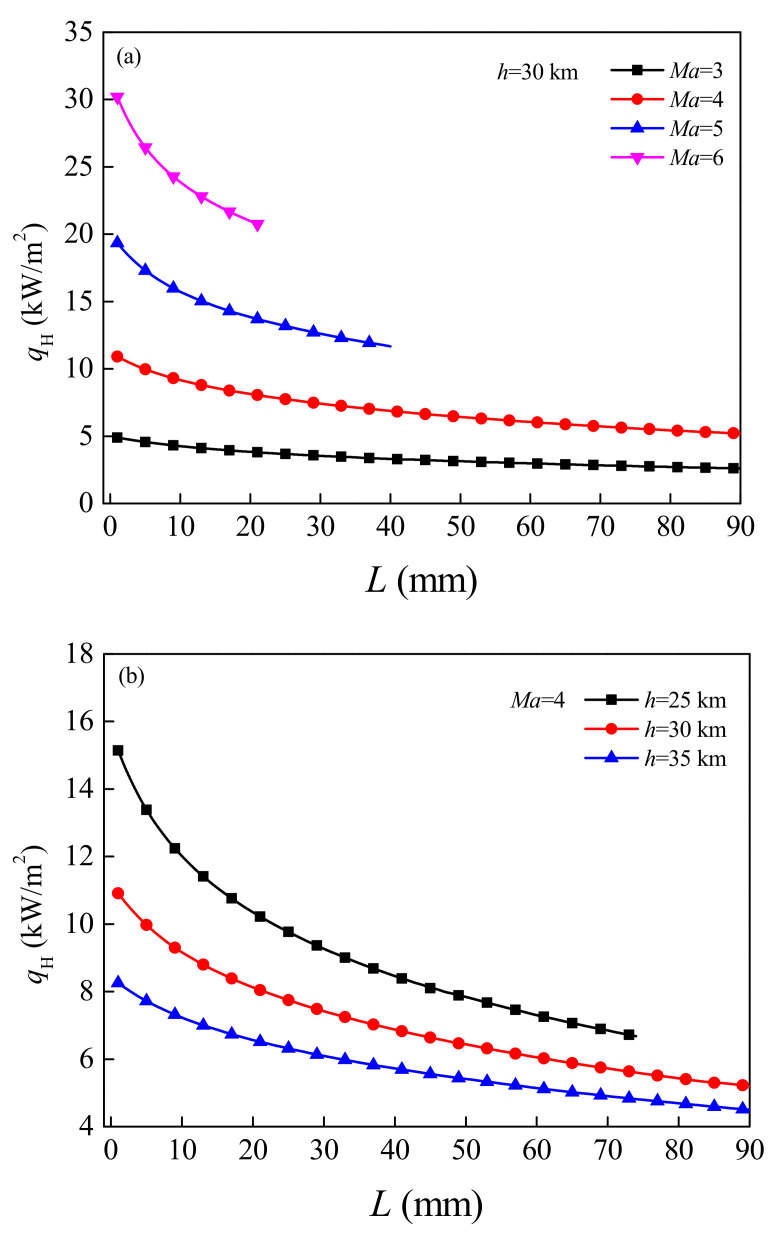
Variation of the heat absorption of the hot end of the TEG changing with the length of the TE legs under different flight Mach numbers (**a**) and flight altitudes (**b**).

Figure 6 shows the variation law of heat flux density *q*_H_ (i.e., aerodynamic heat flux density) absorbed by the hot end of the TEG from the vehicle wall with the length *L* of the TE leg under different flight Mach number *Ma* and flight altitude *h*. It can be seen that, the heat flux decreases monotonically with the increase of the length of the TE legs, and the tendency of the change becomes more significant with the increase of the Mach number (or the decrease of the flight altitude). Which mainly because the higher the Mach number, the more heat the fuselage generates by rubbing against the air. In addition, the higher the plane flies, the thinner the air, the less heat it produces. Increasing the length of the TE legs will cause the rise of the hot end temperature of the TEG, but the increase of the internal resistance of the TEG and the decrease of the thermal conductivity will weaken the influence of the hot end temperature on the heat absorption of the TEG. For flight conditions *Ma* = 6, *h* = 30 km, when the length of TE legs increases from 1 mm to 21 mm, the corresponding heat flux decreases from 30.186 kW/m^2^ to 20.746 kW/m^2^. For flight conditions *Ma* = 4, *h* = 25 km, with the length of TE legs increasing from 1 mm to 74 mm, the corresponding heat flux decreases from 15.14 kW/m^2^ to 6.682 kW/m^2^.

**Figure 7:**
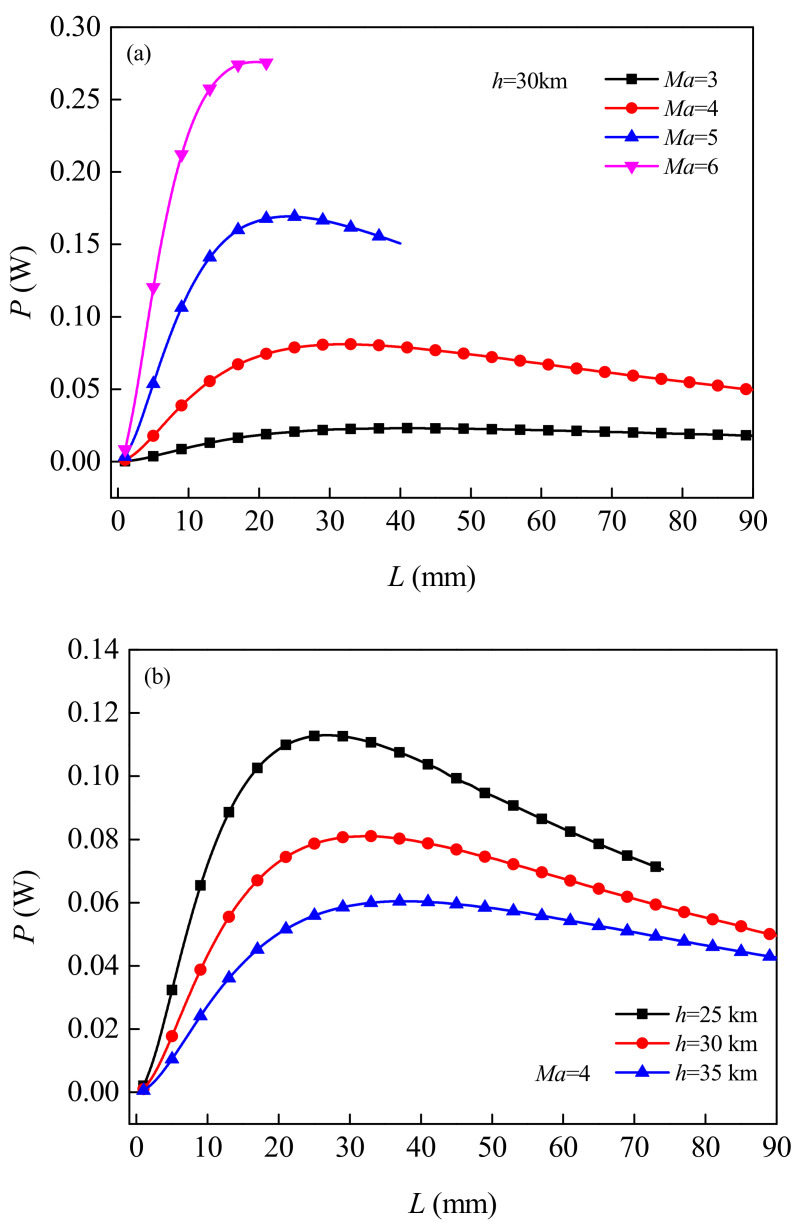
Variation of output power of TEGs changing with the length of the TE leg under different flight Mach Numbers (**a**) and flight altitudes (**b**).

Figure 7 shows the change law of the output power *P* changing with the length *L* of the TE leg under different flight Mach Numbers *Ma* and flight altitude *h*. As can be seen from the Figure, in the set operating condition, the higher the flight Mach number (or the lower the flight altitude), the higher the output power. Since the more serious aerodynamic heating phenomenon of the vehicle, the greater the temperature difference between the two ends of the TEG, resulting in higher output voltage of the TEG. It can also be observed from the Figure that, with the increase of the length of TE legs, the output power first increases and then decreases. When the output power reaches its maximum, the internal resistance of the TEG is equal to the external load resistance. In addition, there is an optimal length for the TE legs. If the given flight attitude is *h* = 30 km, for the flight Mach number *Ma* = 3, 4, 5, 6, the optimal length for the TE legs is *L* = 41, 32, 24, 19 mm, respectively, and the maximum output power is *P* = 0.023, 0.081, 0.169, 0.276 W, respectively. When the flight Mach number is *Ma* = 4, for the flight altitude *h* = 25, 35 km, the optimal length of the TE leg is *L* = 27, 38 mm, and the corresponding maximum output power is *P* = 0.113, 0.06 W, respectively. 

**Figure 8:**
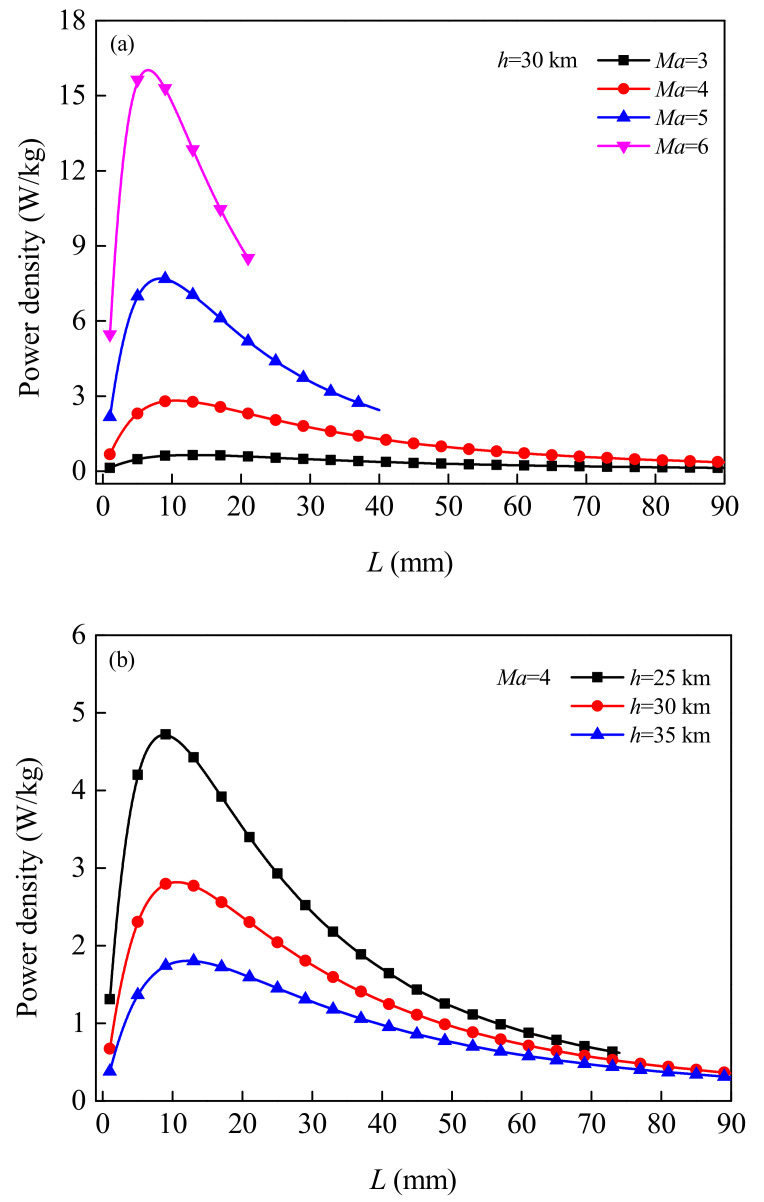
Variation of output power density of TEGs changing with the length of the TE leg under different flight Mach Numbers (**a**) and flight altitudes (**b**).

Furthermore, the predicted results of power density are given in Figure 8. The power density is defined as the ratio of the output electrical power to the mass of the TEG. Similarly, with the increase of the length of the TE legs, the power density first increases and then decreases. At high Mach Numbers (or low flight altitudes), the change is even more pronounced. For *h* = 30 km and under the condition *Ma* = 3, 4, 5 and 6, the length of the TE legs corresponding to the maximum power density is *L* = 13, 11, 8, and 7 mm, respectively; For *Ma* = 4, the corresponding length of TE legs under *h* = 25 km and 35 km is *L* = 9 mm and 12 TEGs, mainly due mm, respectively. The results show that the maximum output power and the maximum power density cannot be obtained with the same length of the TE leg. Which mainly related to the internal resistance of to the temperature dependence of the resistivity of thermoelectric materials.

**Figure 9:**
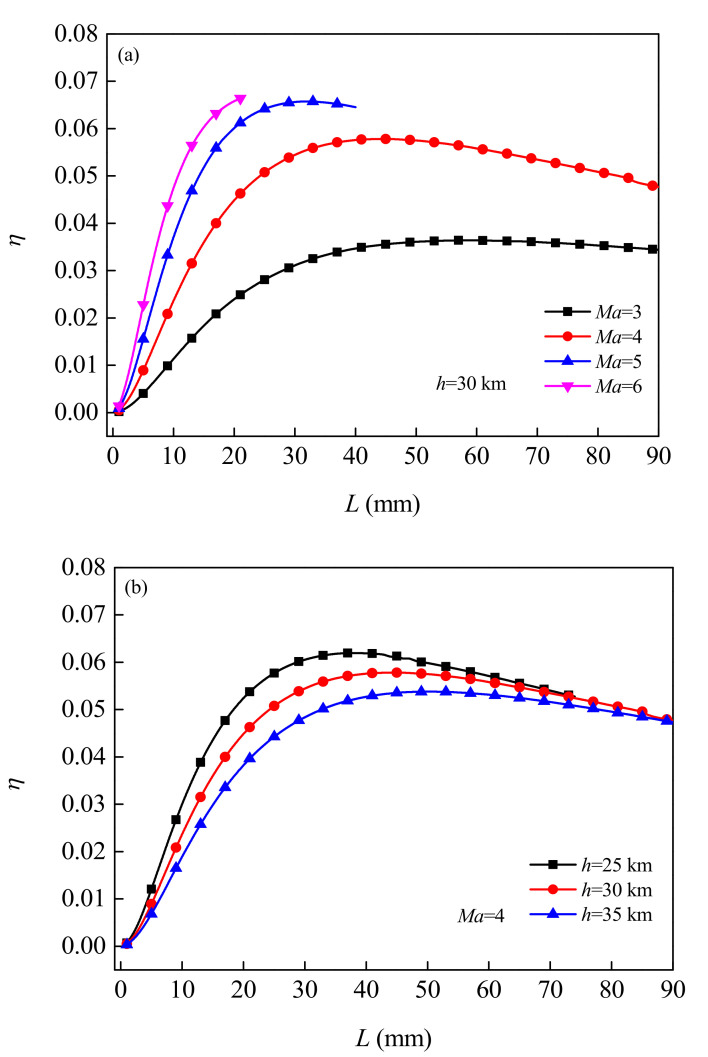
Variation of the conversion efficiency of TEGs changing with the length of the TE leg under different flight Mach numbers (**a**) and flight altitudes (**b**).

Figure 9 shows the variation law of TE conversion efficiency (heat utilization ratio) *η* changing with the length *L* of TE leg under different flight Mach number *Ma* and flight altitude *h*. It can be seen that, with the increase of the length of the TE legs, the conversion efficiency first increases and then decreases. Which mainly related to the change of internal resistance. For flight conditions *Ma* = 6, *h* = 30 km, and when the length of TE legs is *L* = 21 mm, the energy recovery system can achieve the maximum conversion efficiency of 6.636%. For flight conditions *Ma* = 3, *h* = 30 km, and when TE leg length is *L* = 59 mm, the maximum conversion efficiency is 3.639%. As can be seen from Figure 7 and Figure 9, in the same flight condition, the length of the TE leg corresponding to the maximum conversion efficiency is not equal to the length of the TE leg corresponding to the maximum output power. In the practice, the matching length of TE leg should be selected according to different application requirements. The results show that increasing the flight Mach number and decreasing the flight altitude (within the reference value range in this paper) will cause the thermal flux, temperature difference and material properties of the TEG to change, thus contributing to the improvement of the output performance of the energy recovery system.

## 4. Conclusions

This paper proposes a scheme for recovering aerodynamic heat of a vehicle using Bismuth-Telluride-based TEGs, and a mathematical model of the TE conversion behavior of the system is established, obtaining the influence rules of typical flight conditions and geometric parameters of TE structure on the TE conversion performance of the energy recovery system. The main conclusions are obtained as follows:

1. With the increase of the length of the TE legs, the temperature of the hot end of the TEG increases and the heat absorbed decreases, while the output power, power density and conversion efficiency all increase first and then decrease.
2. In practical application, appropriate length of thermoelectric leg should be selected according to specific service conditions and external load.
3. When Ma = 6, h = 30 km, L = 21 mm, the maximum conversion efficiency of energy recovery system can go up to 6.636%.
4. When flight Mach number is higher and flight altitude is lower, the output performance of energy recovery system can be improved obviously.

This paper provides ideas for the recovery and utilization of aerodynamic heat and energy refinement management of vehicle, but this paper is only a preliminary exploration of the application of TEGs in supersonic aircraft, and still stays at the stage of theoretical analysis. Further research and experiments are needed to realize its practical application.

## Nomenclature

C	heat capacity, J kg^−1^ k^−1^
E	electric potential, V
h	flight altitude, km
I	current, A
J	current density, A m^−2^
L	length of the thermoelectric legs, mm
$p_{a}$	airflow pressure, Pa
P	output power of TEGs, W
q	Heat flux density, W/m^2^
Re	Reynolds number of the flow
$R_{0}$	load resistance, Ω
T	temperature, K
$T_{h}$	temperature of hot end, K
$T_{c}$	temperature of cold end, K
α	Seebeck coefficient, V K^−1^
λ	thermal conductivity, W m^−1^ K^−1^
ν	air viscosity coefficient, Pa·s
σ	electric conductivity, S m^−1^
